# Associations between mental health conditions in pregnancy and maternal socioeconomic status: a population-based retrospective cohort study in Ontario, Canada

**DOI:** 10.1186/s12905-024-03499-w

**Published:** 2024-12-23

**Authors:** Qun Miao, Gwyneth Zai, Ian Joiner, Jessica Burnside, Mark Walker

**Affiliations:** 1 Better Outcomes Registry & Network (BORN) Ontario, Children’s Hospital of Eastern Ontario (CHEO), Ottawa, ON Canada; 2 Children’s Hospital of Eastern Ontario Research Institute (CHEO-RI), Ottawa, ON Canada; 3 School of Epidemiology and Public Health, University of Ottawa Faculty of Medicine, Ottawa, ON Canada; 4 Department of Public Health Sciences, Queen’s University, Kingston, ON Canada; 5 General Adult Psychiatry and Health Systems Division, Molecular Brain Sciences Department, Campbell Family Mental Health Research Institute, Centre for Addiction and Mental Health, Toronto, ON Canada; 6 Department of Psychiatry and Institute of Medical Science, Temerty Faculty of Medicine, University of Toronto, Toronto, ON Canada; 7 Clinical Epidemiology Program, Ottawa Hospital Research Institute, Ottawa, ON Canada; 8 Department of Obstetrics & Gynecology, University of Ottawa, Ottawa, ON Canada; 9 Internationalization and Global Health, University of Ottawa, Ottawa, ON Canada; 10 Department of Obstetrics, Gynecology & Newborn Care, The Ottawa Hospital, Ottawa, ON Canada

**Keywords:** Retrospective cohort study, Mother, Pregnancy, Antenatal, Mental health conditions in pregnancy, Depression, Anxiety, Socioeconomic status

## Abstract

**Background:**

The World Health Organization has recognized maternal mental illness as an emerging issue. Previous studies have indicated that maternal mental illness is associated with socioeconomic status (SES). However, there is a lack of research concerning the mental health of pregnant people with low SES in Ontario, Canada. In this study, we examined associations between mental health conditions during pregnancy and two SES indicators: the pregnant person’s residential neighbourhood income and education level.

**Methods:**

A population-based retrospective cohort study was conducted, consisting of all singleton pregnancies resulting in stillbirths or live births in Ontario hospitals from April 1, 2012, to March 31, 2021. Data were linked from the BORN Information System database, Canadian Institute for Health Information Discharge Abstract Database, and Canadian Census. Poisson regression with robust error variance models was performed to estimate the relative risks of anxiety, depression, anxiety and/or depression, or any mental health condition during pregnancy, by SES indicator. We adjusted for maternal age, obesity status in pre-pregnancy, certain pre-existing maternal health conditions, substance use during pregnancy, race, and rural or urban residence.

**Results:**

Within the cohort (*n* = 1,202,292), 10.5% (126,076) and 8.1% (97,135) of pregnant individuals experienced anxiety and depression, respectively, and 15.8% (189,616) had at least one mental health condition during pregnancy. The trend test (*p* < 0.0001) showed a significant downward trend in the total rates of mental health conditions by increasing SES quintiles. Pregnant individuals in the lowest neighbourhood income quintile tended to have a higher risk of anxiety (aRR: 1.24, 95%CI: 1.22–1.27), depression (aRR: 1.56, 95%CI: 1.52–1.59), anxiety and/or depression (aRR: 1.13, 95%CI: 1.11–1.15), or any mental health condition (aRR: 1.18, 95%CI: 1.16–1.19). Similarly, pregnant people living in the lowest education level neighbourhoods had higher likelihoods of anxiety (aRR: 1.66, 95%CI: 1.62–1.69), depression (aRR: 2.09, 95%CI: 2.04–2.14), anxiety and/or depression (aRR: 1.42, 95%CI: 1.39–1.44), and any mental health condition (aRR: 1.41, 95%CI: 1.38–1.43).

**Conclusions:**

Despite a universal healthcare system, the variations in mental health prevalence and risk during pregnancy based on SES suggest health inequity in Ontario, Canada. Future studies are needed to examine the mechanisms of this health inequity to guide policy makers in reducing disparities in Ontario.

**Supplementary Information:**

The online version contains supplementary material available at 10.1186/s12905-024-03499-w.

## Background

Mental health conditions during pregnancy have been recognized as an emerging issue and a major public health challenge. The World Health Organization (WHO) has highlighted the importance of supporting perinatal mental health services on a global scale [1]. During the perinatal period – throughout pregnancy and the postpartum period – mental health disorders are the most common health complication encountered [2, 3]. Prevalence of symptoms of anxiety and depression are estimated to be 15.4% and 9.7%, respectively, amongst pregnant people in Canada [4]. Worldwide antenatal prevalence of anxiety ranged from 1 to 26% [5] with depression prevalence during pregnancy reported as 20.7% (95%CI: 19.4–21.9%) [6]. Mental health conditions during pregnancy affect both the pregnant person and child, in addition to being associated with poorer birth [7, 8], postpartum [9] and early childhood [10] outcomes.

Several studies have found that the prevalence of mental health illnesses in pregnancy is higher among people with lower socioeconomic status (SES) compared to those with higher SES [11–14]. A population-based study conducted in the Netherlands with 5,398 pregnant people found that lower family income increased the risk of antenatal anxiety (β:0.107, 95%CI: 0.040–0.174) and depression (β:0.134, 95%CI: 0.073–0.195) [15]. In a prospective cohort study in Vietnam that included 497 pregnant people, it was demonstrated that those with greatest economic difficulty had increased risk of non-psychotic mental health conditions measured at ≤ 28 gestational weeks of pregnancy (RR:1.3, 95% CI: 1.1–1.7) [16].

Findings are inconsistent amongst studies examining the relationship between SES and antenatal mental health. A systematic review found no association between SES measured by composite scores and antenatal depressive symptoms, however, a bivariate analysis that did not control for confounders demonstrated an association between subcomponents of SES – income and education – with depressive symptoms during pregnancy [17]. Bayrampour et al. [18] conducted a systematic review whereby 5 of 9 studies reported evidence of an association between antenatal anxiety and low income.

There is a need for additional research to examine the effect of SES on risk of maternal mental health conditions since it is unknown if there are health inequities in Ontario, Canada. There may be geographic location-specific conditions that exacerbate the burden of insufficient resources, such as high prevalence of common mental disorders (non-psychotic mental health conditions) in rural Vietnam [16], supporting the need for national and subnational investigations of this relationship. In this study, we examined associations between antenatal mental health conditions and two maternal SES indicators: residential neighbourhood income and education level [19].

## Methods

### Study design

This is a population-based retrospective cohort study of pregnant people who gave birth between April 1st, 2012, to March 31st, 2021, in Ontario, Canada.

### Study population

All pregnant individuals who had singleton live births and stillbirths with a birthweight of ≥ 500 g or a gestational age of ≥ 20 weeks that occurred in Ontario hospitals were included in the study cohort. We excluded terminations, pregnant persons who did not reside in Ontario, Canada as well as those with multiple gestational births. In total, 68,602 records were excluded. The final cohort consisted of 1,202,292 pregnant individuals.

### Data sources and linkage

#### Better Outcomes Registry & Network (BORN)

BORN is a prescribed pregnancy and birth registry in Ontario, Canada that collects data via the BORN Information System (BIS) on all hospital births in the province. The births captured in the BIS represent > 99% of the births that occur in Ontario, making BORN the largest perinatal registry in Canada [20]. Alongside data on births, BORN also gathers information on pregnancy and newborns that can be accessed in aggregate form for population-level research [20]. In an external audit, Public Health Ontario concluded high accuracy of the BORN data based on alignment with literature and other databases [21]. BORN maintains high quality data using multiple strategies including routine quality tracking and reporting, data quality evaluations and audit [22, 23], and formal training sessions for individuals entering data [24].

#### Canadian Institute for Health Information (CIHI)

On a yearly basis, pregnancy records from the Discharge Abstract Database (DAD) – maintained by CIHI – are provided to BORN. Ontario’s acute care hospital discharge information is accessible in the DAD [25], allowing for identification of those who had a psychiatric diagnosis during pregnancy.

#### 2016 Canadian Census

The variables of neighbourhood education and income from the 2016 Census of the Canadian population were utilized in our study [26].

#### Postal Code Conversion file plus (PCCF+) version 7D

The PCCF + is a SAS program accompanied by Statistics Canada datasets that contain postal codes matched to census geographic areas [26–28]. The PCCF + program has procedures to address issues with partial postal codes as well as discrepancies between postal and census boundaries [28]. We used the PCCF + to link maternal residence to neighbourhood SES indicators from the 2016 Census.

#### Data linkages

**Fig. 1:**
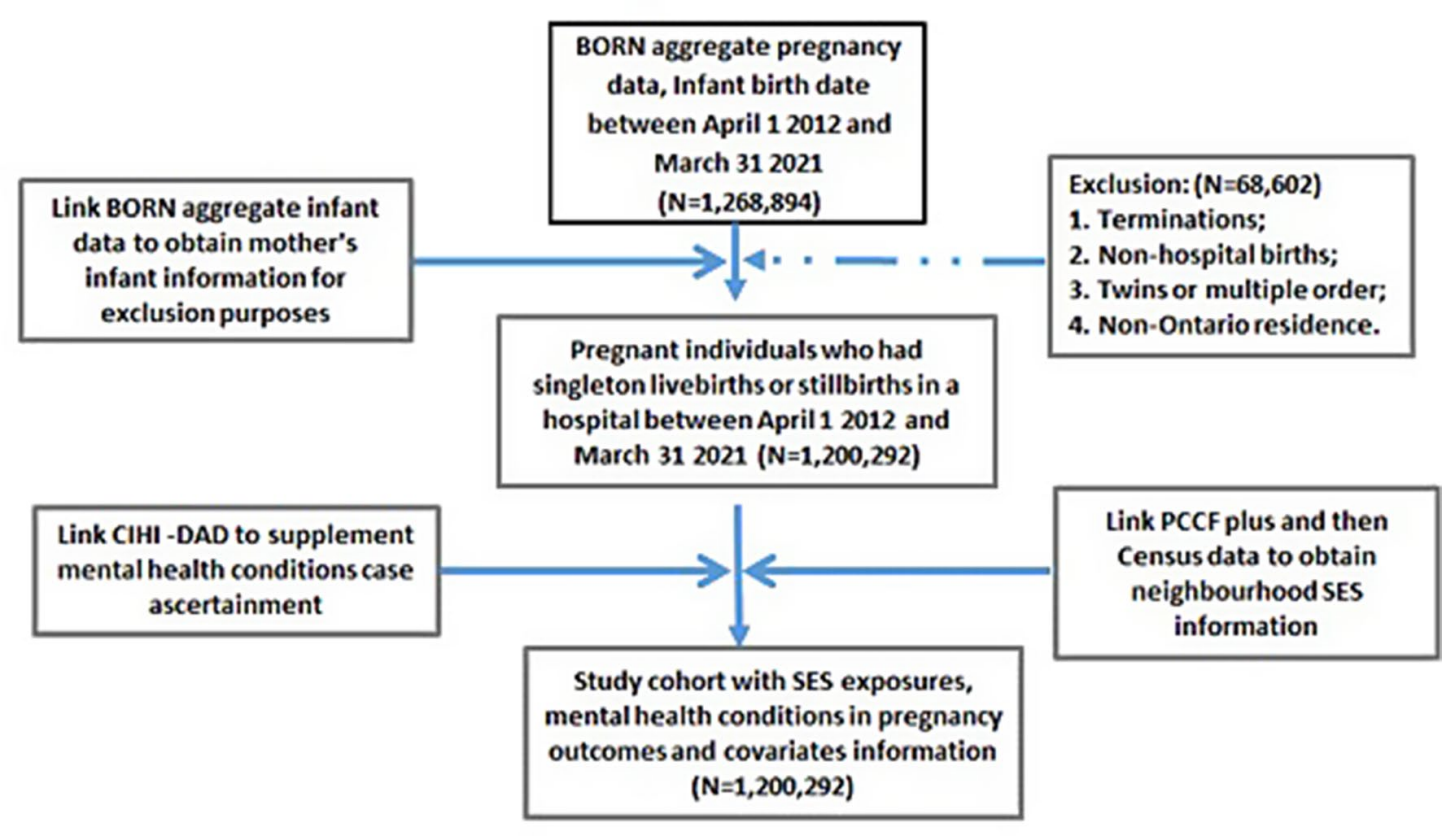
Flowchart of study cohort

We first extracted a BIS dataset with aggregate pregnancy data limited to date of births within the study timeframe, and then linked it to a BIS aggregate infant dataset which allowed for exclusion of records that did not meet the inclusion criteria. Linkage with CIHI-DAD, PCCF+, and the 2016 Census generated the final cohort for analysis (See Fig. 1).

### Exposure measurement

In this study, maternal SES is the exposure. Due to data limitations, we were not able to estimate SES indicators on the individual level. Instead, we linked the study cohort to the Canadian Census data. Two neighbourhood SES indicators were investigated separately: (a) neighbourhood income and (b) neighbourhood education level. Neighbourhood income was estimated using after tax median family income adjusted by household size and categorized into quintiles based on each census metropolitan area or census agglomeration area in Ontario [29].

Neighbourhood education level was estimated by the percentage of the adult population aged 25 to 64 years with a university degree or higher at the dissemination area (DA) level and was categorized by quintiles by ranking all DAs in Ontario [30]. The least value is quintile 1 (Q1) and the greatest value is quintile 5 (Q5).

### Outcome measurement

The outcomes were defined as anxiety (yes, no), depression (yes, no), anxiety and/or depression (yes, no), and any mental health conditions (yes, no) in pregnancy. The BIS and CIHI-DAD database were accessed to retrieve data on self-report or clinical diagnosis of these outcomes. The BIS retrieval included any maternal mental health concerns during this pregnancy including those pre-existing, diagnosed during pregnancy or active during pregnancy [31]. The picklist values for mental health conditions in the BIS registry data were based on both self-report symptoms and/or clinical diagnoses [31]. Due to data limitations, we were not able to differentiate symptoms, disorders or conditions that were pre-existing only, diagnosed during pregnancy or active during pregnancy clearly. In the CIHI-DAD dataset, mental health conditions were identified by the International Statistical Classification of Diseases and Related Health Problems, 10th Revision, Canadian adaptation (ICD-10-CA) codes [32]. The ICD-10-CA codes for any mental health conditions during pregnancy included F00-F99 (Mental and behavioural disorders). The diagnosis codes for anxiety include F06.4 (Organic anxiety disorder), F40 (Phobic anxiety disorders), and F41 (Other anxiety disorders). The diagnosis codes for depression include: F32 (Depressive episode), F33 (Recurrent depressive disorder), and F34.1 (Dysthymia). For the outcome of anxiety and/or depression and the outcome of any mental health conditions, in addition to the cases identified from BIS and DAD, we also included pregnant people with selective serotonin reuptake inhibitor (SSRI) medication use during pregnancy identified via the BORN data source.

### Covariates

Covariates were selected for inclusion in the model based on the findings from the literature review and consideration of the distribution of the covariates within the cohort [13]. Substance use during pregnancy was not in the models for the outcomes of anxiety and depression due to a limit of its range for some observations. The covariates controlled for included maternal age (< 35 years old vs. ≥35 years old), obesity status in pre-pregnancy (yes, no, unknown), pre-existing maternal health conditions including hypertension, diabetes, heart disease and pulmonary disease (yes vs. no), substance use during pregnancy (yes vs. no), race (White, other races, unknown) and rural/urban residence (Yes, rural residence vs. No, urban residence).

### Statistical analysis

Descriptive analyses were performed to show the distributions of anxiety, depression, anxiety and/or depression, and any mental health condition by SES quintiles. The Cochran-Armitage trend test was used to test the prevalence trends of mental health outcomes by SES. Poisson regression with robust error variance models was performed to estimate the relative risk (RR) of the mental health outcomes explored by the pregnant individual’s residential neighbourhood income and education level. For the regression analysis, we excluded pregnant people (*n* = 140,486) with singleton births, stillbirths, or pregnancy terminations after the COVID-19 pandemic (February 28, 2020). We adjusted for maternal age, obesity status in pre-pregnancy, certain pre-existing health conditions (e.g., diabetes mellitus, hypertension, pulmonary disease, and heart disease), substance use during pregnancy, rural residence, and maternal race. We categorized “Unknown” values of obesity and race as a separate level in the multivariable regression analysis. Records with missing values from other variables were excluded in multivariable regression analysis. Sensitivity analysis was conducted by including pregnant people with singleton births, stillbirths, or pregnancy terminations that occurred after February 28, 2020, to evaluate the impact of the COVID-19 pandemic on the associations between mental health conditions during pregnancy and maternal SES. All analyses were performed using SAS 9.4 (SAS Institute Inc., Cary NC) [33].

## Results

In total, we found that 10.5% (*n* = 126,076) and 8.1% (*n* = 97,135) of individuals experienced anxiety and depression respectively, for a total of 14.4% (*n* = 173,061) of individuals with symptoms or clinical diagnosis of anxiety and/or depression during pregnancy. In this study population, 15.8% (*n* = 189,616) had at least one mental health condition or experienced any mental health symptoms during pregnancy.

In this cohort, the majority were under the age of 35 (76.0%); 43.1% of pregnant individuals were nulliparous (parity = 0), and 12.6% resided in a rural area (See Table 1). Prior to pregnancy, approximately 17.5% of individuals were obese and 6.8% had at least one of the following pre-existing health conditions: diabetes mellitus, hypertension, pulmonary disease, or heart disease. Around 39.9% of pregnant individuals were White. Additionally, 11.7% of pregnant individuals reported smoking, social drug use, or alcohol consumption during pregnancy.

**Table 1:** Characteristics of study population (April 1 2012 to March 31 2021)

Variable	Number of Participants	Percentage of Cohort
**Maternal age at birth in years**		
< 35	911,726	76.0
≥ 35	288,562	24.0
Missing	< 6	-
**Maternal obesity in pre-pregnancy**		
No (BMI < 30 kg/m^2^)	848,679	70.7
Yes (BMI ≥ 30 kg/m^2^)	209,822	17.5
Unknown	141,791	11.8
**Maternal race**		
White	478,798	39.9
Asian	219,659	18.3
Black	55,669	4.6
Other	49,216	4.1
Unknown	396,950	33.1
Assisted reproductive technology derived conception*		
No	1,159,178	96.6
Yes	41,114	3.4
**Parity**		
0	516,933	43.1
≥ 1	671,473	55.9
Missing	11,886	1.0
**Pre-existing maternal health condition** ^**€**^		
No	1,118,826	93.2
Yes	81,466	6.8
**Smoking, social drug use, or alcohol consumption during pregnancy**		
No	1,036,427	86.4
Yes	140,147	11.7
Missing	23,718	2.0
**Maternal rural residence**		
No	1,033,147	86.1
Yes	151,341	12.6
Missing	15,804	1.3

*: Missing values of assisted reproductive technology derived conception were combined with “No”

€: Pre-existing health conditions include diabetes mellitus, hypertension, pulmonary disease and heart disease

**Fig. 2:**
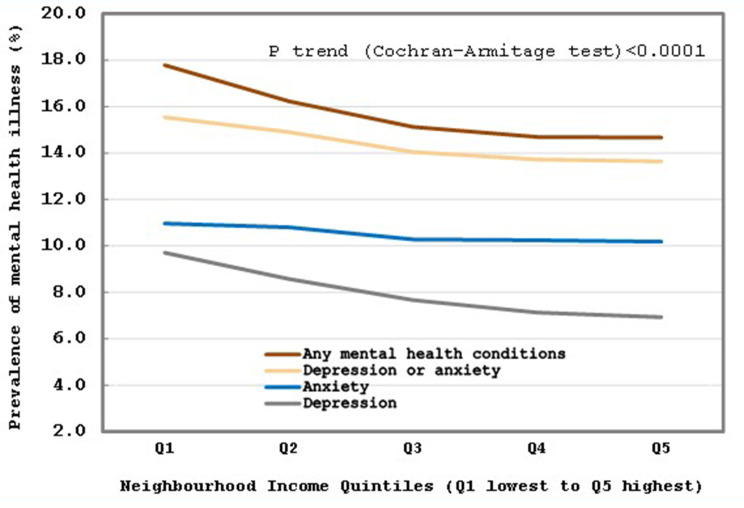
Trend of mental health conditions in pregnancy by quintiles of neighbourhood income (April 1, 2012 – March 31, 2021). *Notes*: Prevalence rates of any mental health conditions, depression and/or anxiety, depression, or anxiety during pregnancy (including pre–existing, diagnosed during pregnancy or active during pregnancy from BORN or CIHI DAD data sources) by neighbourhood income quintiles (Q1 least value to Q5 highest value)

**Fig. 3:**
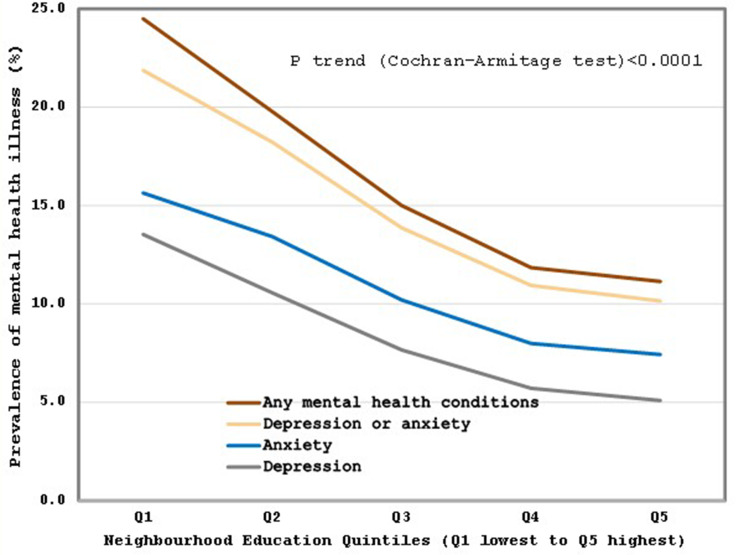
Trend of mental health conditions in pregnancy by quintiles of neighbourhood education level (April 1, 2012 – March 31, 2021) *Notes* Prevalence rates of any mental health conditions, depression and anxiety, depression, or anxiety during pregnancy (including pre–existing, diagnosed during pregnancy or active during pregnancy from BORN or CIHI DAD data sources) by neighbourhood education quintiles (Q1 least value to Q5 highest value)

The Cochran-Armitage trend test showed a significant downward trend for all mental health outcomes assessed along increasing neighbourhood income quintiles (See Fig. 2) and education quintiles (See Fig. 3). Specifically, as income and education quintiles increased (from the lowest to highest), the Cochran-Armitage trend tests for prevalence of (i) anxiety, (ii) depression, (iii) anxiety and/or depression, and (iv) any mental health condition were all significantly decreasing (*p* < 0.0001).

The associations between the SES indicators and mental health outcomes were assessed separately for quintiles 1, 2, 3, and 4 compared with quintile 5 (See Table 2). Pregnant individuals in the lowest neighbourhood income quintile tended to have a higher risk of anxiety (aRR: 1.24, 95% CI: 1.22–1.27), depression (aRR: 1.56, 95% CI: 1.52–1.59), anxiety and/or depression (aRR: 1.13, 95% CI: 1.11–1.15), or any mental health condition (aRR: 1.18, 95% CI: 1.16–1.19) when compared to pregnant persons living in the highest income neighbourhoods. Similarly, pregnant people living in the lowest education level neighbourhoods tended to have higher likelihoods of anxiety (aRR: 1.66, 95% CI: 1.62–1.69), depression (aRR: 2.09, 95% CI: 2.04–2.14), anxiety and/or depression (aRR: 1.42, 95% CI: 1.39–1.44), and any mental health condition (aRR: 1.41, 95% CI: 1.38–1.43) compared to those living in the highest education level neighbourhoods. Increased risk of mental health outcomes for pregnant people in lower income/education neighbourhoods were also consistently observed when comparisons were made between quintile 2 and 5 as well as quintile 3 and 5. After excluding individuals who gave birth after February 28 2020, the prevalence estimates were as follows: anxiety (9.8%), depression (7.9%), anxiety and/or depression (13.8%), and any mental health conditions (15.2%). The Cochran-Armitage trend test showed a similar downward trend of mental health outcomes during pregnancy by SES indicators (See Appendix – Fig. 1a and b). For the analysis of the association between SES and mental health conditions, the multivariable regression results were similar when including pregnant people who gave birth after February 28, 2020 (COVID-19 Pandemic) (See Appendix – Table a).

**Table 2:** Associations between maternal SES and mental health conditions

Outcome	SES Variable	Crude RR			Adjusted RR		
		Crude RR	Lower 95%CI	Upper 95%CI	Adjusted RR	Lower 95% CI	Upper 95% CI
**Mental health conditions**	**Neighbourhood education level**						
	Q1 vs. Q5	2.21	2.18	2.24	1.41	1.38	1.43
	Q2 vs. Q5	1.78	1.75	1.80	1.30	1.28	1.32
	Q3 vs. Q5	1.35	1.33	1.38	1.16	1.14	1.18
	Q4 vs. Q5	1.07	1.05	1.09	1.04	1.03	1.06
	**Neighbourhood income**						
	Q1 vs. Q5	1.22	1.21	1.24	1.18	1.16	1.19
	Q2 vs. Q5	1.11	1.09	1.13	1.1	1.08	1.11
	Q3 vs. Q5	1.04	1.02	1.05	1.05	1.03	1.06
	Q4 vs. Q5	1	0.98	1.01	0.99	0.98	1.01
**Anxiety and/or depression**	**Neighbourhood education level**						
	Q1 vs. Q5	2.16	2.13	2.19	1.42	1.39	1.44
	Q2 vs. Q5	1.80	1.77	1.83	1.33	1.31	1.35
	Q3 vs. Q5	1.37	1.35	1.39	1.18	1.16	1.20
	Q4 vs. Q5	1.09	1.07	1.10	1.05	1.04	1.07
	**Neighbourhood income**						
	Q1 vs. Q5	1.15	1.13	1.17	1.13	1.11	1.15
	Q2 vs. Q5	1.1	1.08	1.11	1.1	1.08	1.12
	Q3 vs. Q5	1.04	1.02	1.05	1.05	1.04	1.07
	Q4 vs. Q5	1	0.99	1.02	1	0.99	1.02
**Depression**	**Neighbourhood education level**						
	Q1 vs. Q5	2.65	2.59	2.71	2.09	2.04	2.14
	Q2 vs. Q5	2.08	2.03	2.13	1.74	1.70	1.78
	Q3 vs. Q5	1.52	1.49	1.56	1.39	1.36	1.43
	Q4 vs. Q5	1.13	1.11	1.16	1.12	1.10	1.15
	**Neighbourhood income**						
	Q1 vs. Q5	1.39	1.36	1.42	1.56	1.52	1.59
	Q2 vs. Q5	1.23	1.2	1.26	1.32	1.29	1.35
	Q3 vs. Q5	1.1	1.08	1.13	1.16	1.13	1.19
	Q4 vs. Q5	1.02	1	1.05	1.03	1	1.05
**Anxiety**	**Neighbourhood education level**						
	Q1 vs. Q5	2.10	2.06	2.14	1.66	1.62	1.69
	Q2 vs. Q5	1.80	1.77	1.84	1.50	1.47	1.53
	Q3 vs. Q5	1.37	1.35	1.40	1.26	1.23	1.29
	Q4 vs. Q5	1.08	1.06	1.10	1.08	1.06	1.10
	**Neighbourhood income**						
	Q1 vs. Q5	1.08	1.06	1.1	1.24	1.22	1.27
	Q2 vs. Q5	1.06	1.04	1.08	1.16	1.13	1.18
	Q3 vs. Q5	1.02	1	1.04	1.08	1.06	1.1
	Q4 vs. Q5	1	0.98	1.02	1.02	1	1.04

*Notes* (1) Neighbourhood education level was defined as percentage of people aged 25–64 who had a university degree or higher at a dissemination area; (2) Neighbourhood income was defined as household income for each census metropolitan area/agglomeration; (3) Neighbourhood education level and income were categorized as quintiles. The least value is Q1 and highest value is Q5; (4) Two SES variables were in separate Poisson regression with robust error variance models while adjusting for maternal age, obesity status in pre-pregnancy, pre-existing maternal health conditions, substance use during pregnancy, race and maternal rural residence. (5) Mental health conditions include depression and/or anxiety and other mental health conditions

## Discussion

In summary, we found that 15.8% (189,616) of pregnant individuals in our cohort had a mental health condition during pregnancy; 10.5% (126,076) and 8.1% (97,135) experienced anxiety and depression, respectively. There was a downward trend in the prevalence of mental health conditions during pregnancy from Q1 (lowest SES) through Q5 (highest SES) for both neighbourhood indicators studied. Those living in neighbourhoods classified with the lowest income and education quintiles are at an increased risk of mental health disorders during pregnancy. This relationship persisted when examining anxiety, depression, anxiety and/or depression, or any mental health condition.

There have been published studies to examine associations between maternal SES and postnatal mental health conditions. However, the research regarding antenatal mental health is lacking. Our study brought attention to identifying the pregnant people at risk of antenatal anxiety and depression. Maternal prenatal mental illness may impact maternal physical health and fetus development negatively, such that targeted early interventions could be impactful for improving health and wellbeing. At present, Canada lacks national perinatal mental health screening guidelines, with wait times for mental health care during this life period frequently longer than 2 months [2].

The prevalence of anxiety during pregnancy identified in our study is within the range highlighted in a recent literature review focused on European countries, ranging from 7.7% in Poland to 36.5% in Italy [34]. Our reported prevalence rate of depression (8.1%) during pregnancy was similar with another recent Canadian study (9.7%) [4] but below the range of 15-65% presented in an umbrella review with international data [35]. In Northern Ireland, a population-based study reported that 18.9% of pregnant individuals had a history of at least one mental disorder [36], compared to 15.8% in our study. However, identifying mental health conditions through acute care hospitals or self-report may have resulted in lower estimates in our study. Due to a lack of studies regarding antenatal mental health conditions from population-based studies in Canada, it is unclear if prevalence was underreported in our study.

The gradient of mental health condition prevalence across neighbourhood SES demonstrated in our research is consistent with publications that have also used neighbourhood level indicators [13, 37]. In the Canadian context, a study conducted in the province of British Columbia using administrative data found a clear gradient of decreasing diagnoses of depression as well as anxiety and/or depression with increasing neighborhood income quintiles [13]. In addition, the anxiety alone curve was quite stable for the income quintiles, which is somewhat in line with the British Columbia study, where anxiety may not be as strongly associated with low SES as some other mental health disorders [13]. Beyond Canada, a population-based study in the United Kingdom (UK) found that the prevalence of antenatal depression, anxiety, and serious mental illness increased with greater neighborhood socioeconomic deprivation [37]. The test for trend based on neighborhood socioeconomic deprivation was significant for the odds of antenatal depression, however the odds of antenatal anxiety only showed this trend for specific age groups [37]. These results in the UK align with those reported in our study.

We interpret our results relating to living in lower SES neighborhoods as a proxy for the individual SES. Therefore, comparison to studies of individual SES and adverse mental health conditions during pregnancy can also be informative. In a South African study of 726 pregnant people, lower SES was found to increase the risk of antenatal depression (Crude OR: 2.3, 95%CI: 1.3-4.0), with individual level SES measured using a composite score divided into quartiles [39]. In Italy, a fivefold reduction in the odds of antenatal depression was associated with high economic status (OR: 0.23, 95%CI: 0.10–0.54), while results based on maternal individual education level or employment status were not significant [40]. A prospective cohort in Finland demonstrated a gradient-like effect whereby pregnant women with lower education were more likely to have clinically significant levels of anxiety and depression [38]. Our findings demonstrate an increased risk of mental health outcomes based on both neighbourhood income and education.

Investigations in Australia and the United States found that the COVID-19 pandemic influenced the mental wellbeing of pregnant people [41, 42]. With sensitivity analysis, we found that our prevalence estimates of mental health outcomes during pregnancy were lower after excluding pregnant individuals who gave birth during the COVID-19 pandemic. However, the associations between maternal SES and mental health conditions during pregnancy were not significantly affected by the COVID-19 pandemic.

Despite a universal healthcare system in Canada, our study found mental health inequities for pregnant people in Ontario. While the mechanism is not fully clear, potential explanations are described herein. In general, individuals who experience poverty often lack essential resources such as housing, education, and employment, whereby their quality of life can be compromised and related stress can influence mental health [43]. In an empirical model developed for a path analysis of SES to distress during pregnancy, it was suggested that income both directly and indirectly affects maternal distress, specifically through social support and care in pregnancy [44]. Higher education may affect prenatal distress via health knowledge, coping, economic resources, health status and habits, life events, and/or partner characteristics [38]. More broadly, higher education may have a protective effect in the relationship between adverse childhood experiences and prenatal distress [38]. In this study, we used neighbourhood SES indicators to estimate individual level SES. Lower neighbourhood SES may also imply more disadvantaged and less accessible living environment, which may influence maternal mental health [45].

One of the defining strengths of our study was access to a large provincial level, population-based cohort, which avoids selection bias that could have occurred from direct participant recruitment from various research sites. In addition, this study controlled for many known confounders of maternal SES and mental health during pregnancy including maternal age, race, pre-pregnancy obesity status, pre-existing conditions, substance use, and rural residency. We believe that our findings are generalizable to the Canadian population based on an assumption of sufficient similarity in healthcare and economic context across Canada such that low SES is expected to have a comparable effect on mental health during pregnancy. Despite these strengths, there were also limitations to this study. We used neighbourhood income and education level to estimate SES of individuals due to the limitation of health administrative databases, potentially generating misclassification. However, DAs include 400–700 people and are the smallest standard geographic units in Canada; SES measured at DAs demonstrate a comparable evaluation of risk of health inequities with individual measures of SES [46]. Thus, this method has been widely adopted in large population-based studies when health administrative databases were used [13, 37]. Our report of prevalence of mental health conditions during pregnancy is based on acute medical hospital diagnoses and/or self-report during pregnancy, which may not capture pregnant people receiving mental health care in primary care settings or psychiatric hospitals. We expect that the self-report data may include some diagnoses at locations outside of acute care medical hospitals, but the extent to which this occurred is unknown. Moreover, misclassification of the maternal mental health outcomes may have occurred for three reasons. First, we were not able to differentiate self-report of mild symptoms versus clinical diagnosis of anxiety and depression from the BORN data source. Second, this study focuses on the antenatal depression and/or anxiety and other mental health conditions on the antenatal stage, however due to limitations with the administrative data used, the mental health outcomes of this study include pre-existing mental health conditions. Third, SSRI’s are the most commonly used antidepressants in pregnancy due to their relatively low risk on perinatal outcomes. Thus, we included these records in the category of depression and/or anxiety to best capture depression and/or anxiety. However, we might misclassify panic attacks and post-traumatic stress disorder to major depressive disorders. We plan to improve the data capture in the future studies. Furthermore, we know that mental health conditions can also affect SES, however, we do not expect that given the length of pregnancy, there would be sufficient time for antenatal mental health problems to change SES. Lastly, despite controlling for many confounders, residual confounding remains within our analysis.

Low SES measured at the neighbourhood level via income or education confers an increased risk of mental health disorders during pregnancy. Further investigations of this relationship could be strengthened by directly measuring individual SES, excluding people with pre-existing mental health conditions, and differentiating between mild symptomology and clinically significant disorders. In addition, future studies should explore potential mechanisms and investigate specific characteristics in Ontario, Canada including comparing the most privileged areas with the most marginalised areas using other SES indicators. The WHO highlights that pregnant individuals experiencing poverty may need provision of specific prenatal mental healthcare [1]. Given this evidence of increased burden of disease based on SES, resources and supports targeted at those of low SES as a high-risk group may be helpful in mitigating the harms for pregnant individuals and their children. Increasing focus on perinatal mental health as per US recommendations that clinicians refer high-risk pregnant individuals to counselling services [3] and the difficulties of accessing perinatal mental health services in Canada [2], suggest that action should be timely.

## Conclusions

Despite a universal healthcare system in Canada, an increased risk of mental health conditions during pregnancy is faced by people living in the lowest quintiles of neighbourhood income and education compared to those with highest income and education neighbourhoods. These variations suggest health inequity in Ontario, Canada. In addition, these findings indicate that mental health conditions are common in the antenatal period and targeted, early interventions, especially to those of lower SES, could be effective in improving health and wellbeing.

Future studies are needed to examine the mechanisms of this health inequity to guide policy makers in decreasing disproportionate burden for people with lower SES in Ontario.

## Electronic supplementary material

Below is the link to the electronic supplementary material.

Supplementary Material 1

## Data Availability

The data analyzed for this study are held securely at the prescribed registry, BORN Ontario. Data sharing regulations prevent these data from being made available publicly due to the personal health information in the datasets. Enquiries regarding BORN data must be directed to BORN Ontario (Science@BORNOntario.ca).

## Declarations

### Disclaimer

Parts of this material are based on data and information compiled and provided by CIHI. However, the analyses, conclusions, opinions, and statements expressed herein are those of the author(s), and not necessarily CIHI.

## Abbreviations

SES: Socioeconomic status
DA: Dissemination area
RR: Relative risk
aRR: Adjusted relative risk
CIHI: Canadian Institute for Health Information
DAD: Discharge Abstract Database
ICD-10-CA: International Statistical Classification of Diseases and Related Health Problems, 10th Revision, Canadian adaptation: PCCF+, Postal Code Conversion File Plus
BORN: Better Outcomes Registry & Network
BIS: BORN information system
BMI: body mass index
