# Supplementary material for: Associations between mental health conditions in pregnancy and maternal socioeconomic status: a population-based retrospective cohort study in Ontario, Canada
Source: BMC Womens Health. 2024 Dec 23;24:663. doi: 10.1186/s12905-024-03499-w (PMC11664830; doi:10.1186/s12905-024-03499-w)
Supplement: Supplementary file 1 — Supplementary Material 1 [file 12905_2024_3499_MOESM1_ESM.docx]

**Appendix Sensitivity analysis results**

**Figure 1a. Trend of mental health conditions in pregnancy by quintiles of neighbourhood income (April 1, 2012 – February 28, 2020)**


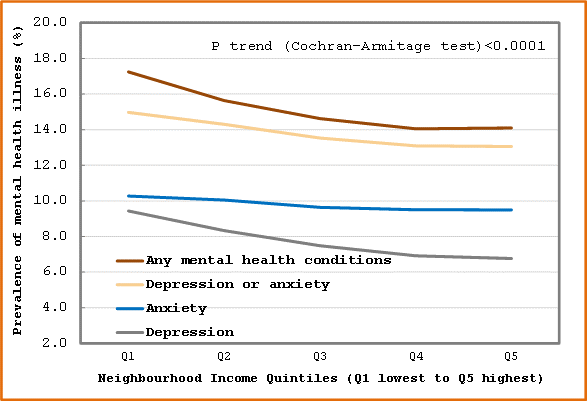


Prevalence rates of any mental health conditions, depression and/or anxiety, depression, or anxiety during pregnancy (including pre–existing, diagnosed during pregnancy or active during pregnancy from BORN or CIHI DAD data sources) by neighbourhood income quintiles (Q1 least value to Q5 highest value).

**Figure 1b.** **Trend of mental health conditions in pregnancy by quintiles of neighbourhood education level (April 1, 2012 – February 28, 2020)**


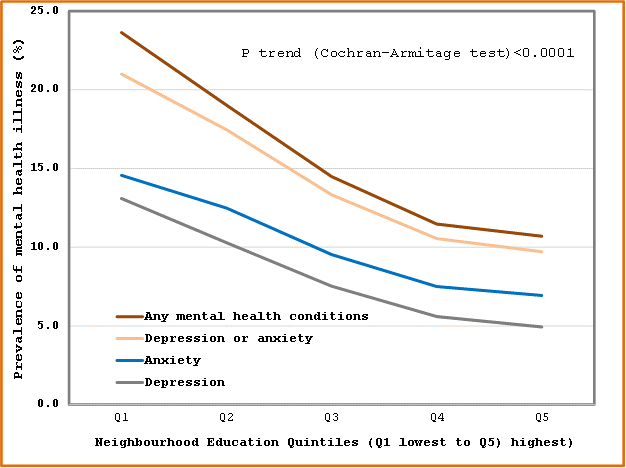


Prevalence rates of any mental health conditions, depression and anxiety, depression, or anxiety during pregnancy (including pre–existing, diagnosed during pregnancy or active during pregnancy from BORN or CIHI DAD data sources) by neighbourhood education quintiles (Q1 least value to Q5 highest value).

**Table a.** **Associations between maternal SES and mental health conditions (April 1 2012 to March 31 2021)**

| **Outcome** | **SES variable** | **Crude RR** | | | **Adjusted RR** | | |
| --- | --- | --- | --- | --- | --- | --- | --- |
|  |  | **Crude RR** | **Lower 95%CI** | **Upper 95%CI** | **Adjusted RR** | **Lower 95% CI** | **Upper 95% CI** |
| **Mental health conditions** | **Neighbourhood education level** |  |  |  |  |  |  |
|  | Q1 vs Q5 | 2.17 | 2.15 | 2.20 | 1.38 | 1.36 | 1.40 |
|  | Q2 vs Q5 | 1.72 | 1.70 | 1.74 | 1.26 | 1.25 | 1.28 |
|  | Q3 vs Q5 | 1.34 | 1.32 | 1.36 | 1.14 | 1.13 | 1.16 |
|  | Q4 vs Q5 | 1.07 | 1.05 | 1.09 | 1.03 | 1.02 | 1.05 |
|  | **Neighbourhood income** |  |  |  |  |  |  |
|  | Q1 vs Q5 | 1.21 | 1.2 | 1.23 | 1.17 | 1.16 | 1.19 |
|  | Q2 vs Q5 | 1.11 | 1.09 | 1.12 | 1.1 | 1.08 | 1.11 |
|  | Q3 vs Q5 | 1.03 | 1.02 | 1.05 | 1.05 | 1.03 | 1.06 |
|  | Q4 vs Q5 | 1 | 0.99 | 1.02 | 1 | 0.99 | 1.01 |
| **Depression or anxiety** | **Neighbourhood education level** |  |  |  |  |  |  |
|  | Q1 vs Q5 | 2.13 | 2.10 | 2.16 | 1.39 | 1.37 | 1.41 |
|  | Q2 vs Q5 | 1.74 | 1.72 | 1.77 | 1.29 | 1.28 | 1.31 |
|  | Q3 vs Q5 | 1.36 | 1.33 | 1.38 | 1.16 | 1.14 | 1.18 |
|  | Q4 vs Q5 | 1.08 | 1.07 | 1.10 | 1.05 | 1.03 | 1.06 |
|  | **Neighbourhood income** |  |  |  |  |  |  |
|  | Q1 vs Q5 | 1.14 | 1.12 | 1.16 | 1.13 | 1.11 | 1.14 |
|  | Q2 vs Q5 | 1.09 | 1.08 | 1.11 | 1.1 | 1.08 | 1.11 |
|  | Q3 vs Q5 | 1.03 | 1.01 | 1.05 | 1.05 | 1.04 | 1.07 |
|  | Q4 vs Q5 | 1.01 | 0.99 | 1.02 | 1.01 | 0.99 | 1.02 |
| **Depression** | **Neighbourhood education level** |  |  |  |  |  |  |
|  | Q1 vs Q5 | 2.63 | 2.58 | 2.69 | 2.05 | 2.01 | 2.10 |
|  | Q2 vs Q5 | 2.00 | 1.96 | 2.04 | 1.66 | 1.62 | 1.70 |
|  | Q3 vs Q5 | 1.51 | 1.47 | 1.54 | 1.37 | 1.34 | 1.40 |
|  | Q4 vs Q5 | 1.13 | 1.10 | 1.15 | 1.11 | 1.09 | 1.13 |
|  | **Neighbourhood income** |  |  |  |  |  |  |
|  | Q1 vs Q5 | 1.4 | 1.37 | 1.43 | 1.56 | 1.53 | 1.59 |
|  | Q2 vs Q5 | 1.24 | 1.21 | 1.26 | 1.33 | 1.3 | 1.36 |
|  | Q3 vs Q5 | 1.11 | 1.08 | 1.13 | 1.16 | 1.14 | 1.19 |
|  | Q4 vs Q5 | 1.03 | 1.01 | 1.05 | 1.03 | 1.01 | 1.06 |
| **Anxiety** | **Neigbourhood education level** |  |  |  |  |  |  |
|  | Q1 vs Q5 | 2.08 | 2.04 | 2.11 | 1.62 | 1.60 | 1.65 |
|  | Q2 vs Q5 | 1.76 | 1.73 | 1.79 | 1.45 | 1.43 | 1.48 |
|  | Q3 vs Q5 | 1.36 | 1.33 | 1.38 | 1.24 | 1.22 | 1.26 |
|  | Q4 vs Q5 | 1.09 | 1.07 | 1.11 | 1.08 | 1.06 | 1.10 |
|  | **Neighbourhood income** |  |  |  |  |  |  |
|  | Q1 vs Q5 | 1.08 | 1.06 | 1.1 | 1.24 | 1.22 | 1.26 |
|  | Q2 vs Q5 | 1.06 | 1.04 | 1.08 | 1.16 | 1.14 | 1.18 |
|  | Q3 vs Q5 | 1.01 | 0.99 | 1.03 | 1.08 | 1.06 | 1.1 |
|  | Q4 vs Q5 | 1.01 | 0.99 | 1.03 | 1.02 | 1 | 1.04 |

Notes: 1) Neighbourhood education level was defined as percentage of people aged 25–64 who had a university degree or higher at a dissemination area; 2) Neighbourhood income was defined as household income for each census metropolitan area/agglomeration; 3) Neighbourhood education level and income were categorized as quintiles. The least value is Q1 and highest value is Q5; 4) Two SES variables were in separate Poisson regression with robust error variance models while adjusting for maternal age, obesity status in pre-pregnancy, pre-existing maternal health conditions, substance use during pregnancy, race and maternal rural residence. 5) Mental health conditions include depression and/or anxiety and other mental health conditions.
